# An Optimization Program to Help Practices Assess Data Quality and Workflow With Their Electronic Medical Records: Observational Study

**DOI:** 10.2196/humanfactors.9889

**Published:** 2018-12-21

**Authors:** Mavis Jones, Reza Talebi, Jennifer Littlejohn, Olivera Bosnic, Jason Aprile

**Affiliations:** 1 OntarioMD Toronto, ON Canada

**Keywords:** chronic disease, electronic medical records, primary care, quality improvement

## Abstract

**Background:**

Electronic medical record (EMR) adoption among Canadian primary care physicians continues to grow. In Ontario, >80% of primary care providers now use EMRs. Adopting an EMR does not guarantee better practice management or patient care; however, EMR users must understand how to effectively use it before they can realize its full benefit. OntarioMD developed an EMR Practice Enhancement Program (EPEP) to overcome challenges of clinicians and staff in finding time to learn a new technology or workflow. EPEP deploys practice consultants to work with clinicians onsite to harness their EMR toward practice management and patient care goals.

**Objective:**

This paper aims to illustrate the application of the EPEP approach to address practice-level factors that impede or enhance the effective use of EMRs to support patient outcomes and population health. The secondary objective is to draw attention to the potential impact of this practice-level work to population health (system-level), as priority population health indicators are addressed by quality improvement work at the practice-level.

**Methods:**

EPEP’s team of practice consultants work with clinicians to identify gaps in their knowledge of EMR functionality, analyze workflow, review EMR data quality, and develop action plans with achievable tasks. Consultants establish baselines for data quality in key clinical indicators and EMR proficiency using OntarioMD-developed maturity assessment tools. We reassessed and compared postengagement, data quality, and maturity. Three examples illustrating the EPEP approach and results are presented to illuminate strengths, limitations, and implications for further analysis. In each example, a different consultant was responsible for engaging with the practice to conduct the EPEP method. No standard timeframe exists for an EPEP engagement, as requirements differ from practice to practice, and EPEP tailors its approach and timeframe according to the needs of the practice.

**Results:**

After presenting findings of the initial data quality review, workflow, and gap analysis to the practice, consultants worked with practices to develop action plans and begin implementing recommendations. Each practice had different objectives in engaging the EPEP; here, we compared improvements across measures that were common priorities among all 3—screening (colorectal, cervical, and breast), diabetes diagnosis, and documentation of the smoking status. Consultants collected postengagement data at intervals (approximately 6, 12, and 18 months) to assess the sustainability of the changes. The postengagement assessment showed data quality improvements across several measures, and new confidence in their data enabled practices to implement more advanced functions (such as toolbars) and targeted initiatives for subpopulations of patients.

**Conclusions:**

Applying on-site support to analyze gaps in EMR knowledge and use, identify efficiencies to improve workflow, and correct data quality issues can make dramatic improvements in a practice’s EMR proficiency, allowing practices to experience greater benefit from their EMR, and consequently, improve their patient care.

## Introduction

Electronic medical record (EMR) adoption among Canadian primary care physicians has grown steadily; 75% now use EMRs, with some provinces—including Ontario—reporting adoption rates >80% [1]. Compared with paper-based practices, EMR-based practices show substantial improvements in population health management [2]. Research investigating the implementation of meaningful use criteria associated with the Health Information Technology for Economic and Clinical Health Act in the United States furthers the argument that care improvements require advanced EMR use. Studies on the quality of diabetes and cancer care in that context suggested that primary care practices need support to redesign work processes with population health management targets in mind [3-6].

However, the benefits of an EMR for patient care and population health cannot be realized unless practices become proficient [7,8], and studies have indicated that practices approaching EMR implementation as a complex change management project would have the greatest success [3,9]. Even in terms of practice management, supports such as workflow optimization [10] and resolution of workarounds [11] are necessary to help a practice realize the full benefit of their EMR.

In Ontario, community-based physicians using a certified EMR have access to OntarioMD’s EMR Practice Enhancement Program (EPEP). The program deploys consultants who provide on-site analysis of a practice’s current EMR proficiency, identify their priorities, and provide recommendations for concrete steps to achieve them. The EPEP was established in early 2016, and at the time of writing this paper had provided in-person support to >1000 clinicians and practice staff.

## Methods

### Electronic Medical Record Practice Enhancement Program

The EPEP is available to all community-based physicians using a certified EMR in Ontario, at no fee, and is promoted to physicians through health care sector conferences and services provided by OntarioMD (eg, regional field staff members who help physicians connect with provincial and local health care information systems). Clinicians often present to the EPEP with the knowledge that their EMR can help them with quality improvement projects but requiring additional knowledge or support on how to get the most from this tool.

### Electronic Medical Record Maturity Model

**Figure 1:**
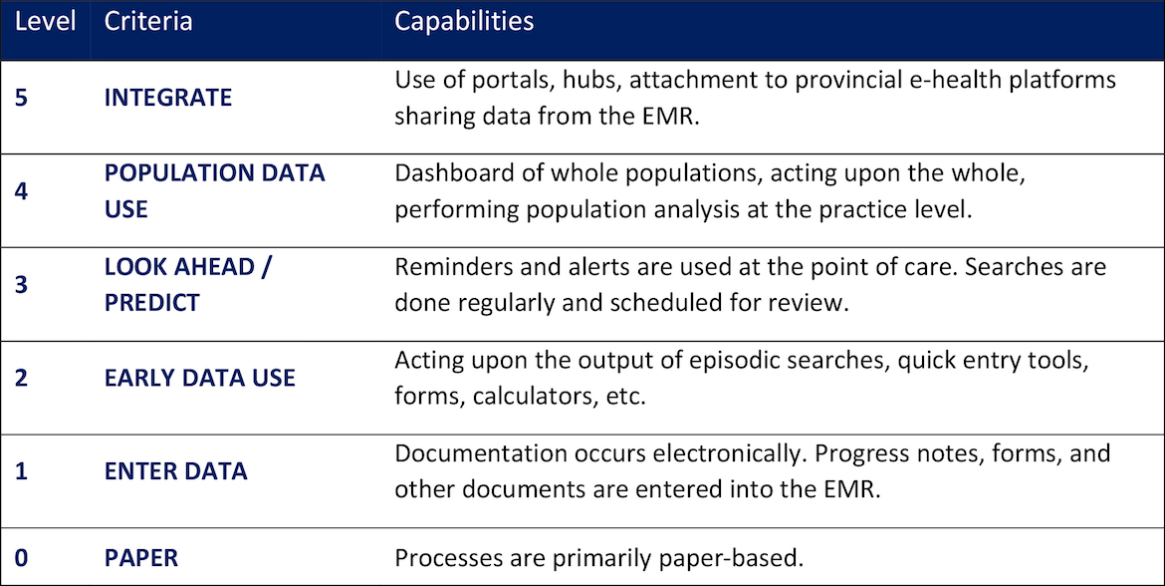
OntarioMD’s electronic medical record maturity model. EMR: electronic medical record.

A foundation of the EPEP method is the EMR Maturity Model (EMM) [12]. The EMM was developed in line with international best practices for measuring the EMR proficiency (eg, the Healthcare Information and Management Systems Society Electronic Medical Record Adoption Model [13]) and validated through engagements with clinical practices. It articulates 6 levels of proficiency ranging from paper-based to fully digital (Figure 1).

### Electronic Medical Record Progress Assessment

**Figure 2:**
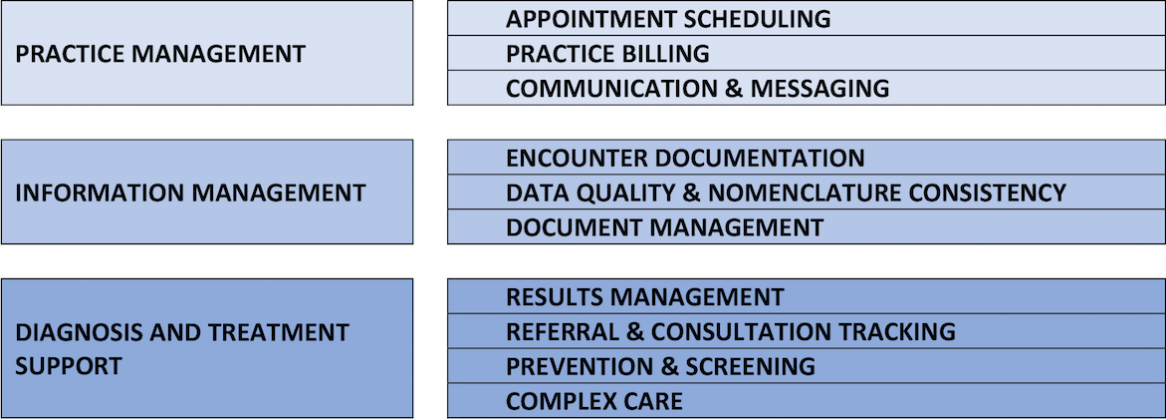
Functional areas and key measures on the EMR (Electronic Medical Record) Progress Assessment.

The EMM provides the foundation for the EMR Progress Assessment (EPA) [14], an Web-based self-assessment tool that allows EMR users to identify their level of proficiency in the functional areas of Practice Management, Information Management, and Diagnosis and Treatment Support, and corresponding key measures (Figure 2).

The EPA is available over the Web [14] to any EMR user who wishes to conduct a self-assessment but is also used within the context of an EPEP engagement. Consultants administer the EPA to assess a practice’s baseline maturity level for each key measure a practice has identified as a priority. Consultants similarly conduct a data quality review (DQR) to establish a baseline for data completeness in areas directly related to the priority key measure (eg, assessing whether blood sugars are recorded for people with diabetes, as associated with complex care/chronic disease management). These baselines and a clinic workflow analysis help consultants develop a diagnostic profile and generate recommended actions targeted at the practice’s goals.

### Electronic Medical Record Practice Enhancement Program Engagement

Generally, an EPEP engagement consists of the following:

- *Current state assessment* using the EPA and a gap analysis to help the practice identify priority areas for improvement;
- *Analysis of data quality and workflow* to determine causes of data discrepancies and establish baselines for specified clinical measures and identify ways to improve the efficiency of workflow;
- *Customized action plan* development that provides concrete, achievable tasks designed to improve data quality in identified practice priority areas and overall practice management;
- *Postengagement evaluation* using the EPA to measure EPEP-driven improvements in EMR data quality and proficiency; depending on the amount of work required in the action plan, postengagement evaluation can be done at 3 or 6 months postbaseline (and again at 12 months to assess the sustainability of improvements).

EPEP consultants tailor their approach according to each practice’s unique characteristics, priorities, and pain points. Each consultant in the program is asked annually to provide a detailed account of one engagement they identified as demonstrating typical challenges faced by primary care practices and how the EPEP method was customized to the practice’s priorities. (Note: the collection of data at the baseline and post encompasses, at least, a year to assess the sustainability of the change, and the program had celebrated its second anniversary at first writing; these examples were selected from a limited cohort, and we look forward to providing further examples in future as the cohort grows.) Three of the authors of this paper (RT, JL, and OB) are consultants who developed the first 3 examples. They selected these from their engagement roster for their distinct priorities and problems, as well as their ability to reflect common challenges. In this paper, we provide a high-level description of the approach taken by consultants, the engagement’s timeline, and additional actions prescribed and taken. The success of any engagement is typically measured by progress against multiple indicators; for this paper, we limit the discussion to indicators on priority areas shared by all 3 practices (and, indeed, representative of primary care practice priorities). Please note that as an artefact of data collection procedures over time, not all n values were available. We elected to omit all for consistency's sake. Requests for more information can be directed to the corresponding author.

Finally, in the discussion section, we contextualize these examples within the larger cohort of EPEP-assessed maturity data, consider limitations to the EPEP approach and our analysis, and discuss next steps for assessing the impact of the program over time.

## Results

### Practice 1

#### Current State Assessment and Priorities

An EPEP consultant met with Dr. A at Practice 1 in April 2016 to discuss his EMR concerns. The physician identified concerns with the amount of time it was taking to search for certain information on a patient such as test results and prior examinations for chronic conditions.

#### Data Quality Review and Workflow Analysis

To review the quality of data captured within Dr. A’s EMR, the consultant ran queries on (1) roster size; (2) preventive care coverage; (3) the number of diabetic patients based on the diagnosis code; (4) the number of diabetic patients based on the diagnosis noted in the problem list; (5) patients with glycated hemoglobin (HbA_1c_) >7 without a diagnosis in the problem list; (6) the number of diabetic follow-up visits based on the billing code; and (7) the smoking status based on the notation in the risk factor section of the cumulative patient profile (CPP).

The consultant’s data analysis revealed that the documentation captured in the EMR was not accurately reflecting Dr. A’s provision of care. For example, there were 44% more patients with a diabetes diagnosis in the problem list than were billed for diabetic visits. Moreover, 9 patients with high HbA_1c_ did not have a diabetes diagnosis indicated in their problem list and showed insufficient clinical visits in the log.

The consultant documented clinic workflow processes, using participant observation, interviews, and activity diagrams. Interviews revealed an onerous workflow problem preventing Dr. A from using the EMR’s diabetic flowsheet feature. The clinic was not receiving laboratory results through the EMR, relying instead on paper, scans, or faxes. As a result, diabetes notes were being manually recorded in the record’s chart section. Dr. A’s clinical documentation was precise and accurate, but searching for patients’ information during visits was inefficient.

#### Action Plan

The EPEP consultant presented Dr. A with their findings from the DQR and workflow analysis and proposed the following action plan:

1. Follow-up with the EMR provider to address technical issues in the transfer of laboratory results.
2. Periodically run and review a cumulative preventive care report.
3. Periodically run reports on patients due for screening or diabetic follow-ups, to ensure critical procedures are tracked.
4. Focus on recording diagnosis in the problem list section.
5. Adopt and adhere to the agreed nomenclature to ensure accuracy of reminders and reports.

#### Postengagement Evaluation

Dr. A executed the action plan, and the consultant provided coaching and reviewed progress. The laboratory interface issue was resolved quickly, and a staff member was assigned to handle the manual download still required for one provider. Dr. A began running diabetic population reports to compare diagnoses and billings. The practice updated charts to resolve discrepancies, reviewed reminders and added new ones, and retooled their workflow to contact diabetic and other patient populations proactively.

With better control over his workload, Dr. A began scheduling follow-ups for screening and diabetes care and set up access for this patient population to self-care supports like nutritional counseling.

In December 2016, the consultant conducted a follow-up review with Dr. A, and postengagement figures were compared with the initial DQR (Table 1).

All indicators are evidence of a positive change in the completeness of documentation, from the most dramatic (cervical cancer screening) to the least (smoking status captured).

### Practice 2

#### Current State Assessment and Priorities

In June 2016, EPEP consultants met with 4 physicians—Drs. B, C, D, and E—working in a group practice, who had been using their EMR for several years. The practice was motivated to engage with the EPEP through a desire to ensure they were providing high-quality care to their patients, including preventive services such as screening.

#### Data Quality Review and Workflow Analysis

To review the quality of data captured within this practice’s EMR, the consultants ran queries on (1) roster size; (2) preventive care coverage; (3) the smoking status based on the notation in the risk factor section of the CPP; (4) the number of patients prescribed diabetic medication with the diabetes diagnosis in the problem list; (5) the number of patients with suspected diabetes based on the HbA_1c_ count with the diagnosis in the problem list; and (7) the number of diabetic follow-up visits based on the billing code.

The DQR revealed variances in each physician’s roster. Investigating further, they found that the clinic had not been aware of the roster capitation reports available from the Ministry of Health and Long-Term Care (MOHLTC) as a resource to monitor enrolled patient populations. The consultants inferred that roster variances were also causing inaccuracies in the reports the clinic generated to identify subpopulations for targeted interventions and confirmed this in reviewing the practice’s preventive care data.

In addition, the DQR showed that the smoking status was not consistently recorded in the CPP, and not all diabetic patients were identified as such in the problem list (despite the presence of diabetic billing codes or diabetic medications prescribed).

#### Action Plan

Consultants met with each physician to review their DQR and current workflows and proposed the following action plan: (1) request MOHLTC roster reports; (2) reconcile roster data; (3) review preventive care data post roster reconciliation; (4) develop prevention and screening management protocols; (5) complete the smoking status in CPP for all patients; (6) onsite and remote coaching on monitoring and tracking diabetes billing codes; and (7) the implementation of a diabetic toolbar within the EMR.

Using the MOHLTC roster reconciliation report, the consultants identified roster discrepancies ranging in variance from 11% to 33%. Keen to improve, the physicians agreed to work on all recommendations to improve roster reconciliation, preventive care screening, and diabetes management.

#### Postengagement Evaluation

After approximately 6 months of work on improving their data quality, the practice reengaged with the consultant to work on prevention and screening and chronic disease management. Pre- and postengagement assessed figures for data quality against these indicators, for each clinician’s patient population, are shown in Tables 2-5.

**Table 1:** Postengagement data quality review—Dr. A.

Data quality indicator	Pre-engagement (May 2016)	Postengagement (December 2016)
Breast cancer screening, %	61.00	65.00
Cervical cancer screening, %	44.29	65.83
Colorectal cancer screening, %	48.79	61.81
Smoking status captured, %	61.35	61.36
Patients with high glycated hemoglobin without diabetes on the problem list, n	10	1

**Table 2:** Postengagement data quality review—Dr. B.

Data quality indicator	Pre-engagement (June 2016)	Postengagement (May 2017)
Breast cancer screening, %	51.97	53.97
Cervical cancer screening, %	49.00	60.00
Colorectal cancer screening, %	35.00	35.02
Smoking status captured, %	2.01	17.02
Patients with high glycated hemoglobin without diabetes on the problem list, n	65	13

**Table 3:** Postengagement data quality review—Dr. C.

Data quality indicator	Pre-engagement (June 2016)	Postengagement (May 2017)
Breast cancer screening, %	64.01	70.00
Cervical cancer screening, %	61.02	68.98
Colorectal cancer screening, %	38.03	41.97
Smoking status captured, %	3.00	18.98
Patients with high glycated hemoglobin without diabetes on the problem list, n	63	20

**Table 4:** Postengagement data quality review—Dr. D.

Data quality indicator	Pre-engagement (June 2016)	Postengagement (May 2017)
Breast cancer screening, %	50.99	55.98
Cervical cancer screening, %	48.00	58.00
Colorectal cancer screening, %	39.03	45.99
Smoking status captured, %	16.03	29.96
Patients with high glycated hemoglobin without diabetes on the problem list, n	4	7

**Table 5:** Postengagement data quality review—Dr. E.

Data quality indicator	Pre-engagement (June 2016)	Postengagement (May 2017)
Breast cancer screening, %	62.99	67.99
Cervical cancer screening, %	65.01	72.99
Colorectal cancer screening, %	34.99	43.99
Smoking status captured, %	47.01	48.99
Patients with high glycated hemoglobin without diabetes on the problem list, n	142	55

As shown, data quality improved against most indicators for all clinicians. The level of improvement depended in part on the baseline assessment. For example, the smoking status was a significant improvement for Drs. B, C, and D. However, Dr. E already had a comparatively high rate of capture for smoking status. Similarly, where the direction of change was opposite from expected—namely, Dr. D’s patients with high HbA_1c_ without diabetes on the problem list—this is still the result of improvement in overall data quality. Notably, identifying the problem allows the practice to correct it.

Consultants continued to coach this group on optimizing their EMR use. They developed and implemented a diabetic toolbar to assist with data entry, display a chronic disease management flowsheet, and provide appropriate information at the point of care. Together with the practice, they implemented a preventive care window and smoking status toolbar to streamline information capture.

### Practice 3

#### Current State Assessment and Priorities

An EPEP consultant met with Dr. F in January 2016. The practice acknowledged they were not consistently entering data into the EMR and recognized that improving data quality and the consistency with which they entered data would help the practice measure the quality of care they provided. In addition, they were looking for guidance on changes to the MOHLTC’s reporting requirements and how to be better prepared for them.

#### Data Quality Review and Workflow Analysis

Based on discussions with Dr. F, the consultant focused on reviewing EMR data associated with preventive care screening and diabetes. The data collection was completed in May of 2016, during which time the consultant conducted EMR queries and produced reports on (1) preventive care; (2) the number of diabetic patients (based on diagnosis code 250); (3) the number of diabetic patients (based on diagnosis noted in the problem list); (4) patients with the last eye exam recorded; and (5) the smoking status (based on the notation in the risk factor section of the CPP).

The consultant conducted interviews with Dr. F and staff to better understand practice workflow and the role of the technology in their clinic to help address their issues.

#### Action Plan

In April 2017, all findings were presented to Dr. F and the clinic manager; as in other examples discussed here, the consultant proposed action plans focused on “quick wins” to improve data quality:

- Review rules for reminders; remove unnecessary ones, and add missing ones (coaching provided).
- Ensure the completion of required activities when a reminder becomes active.
- Periodically run and review a cumulative preventive care report.
- Implement a reminder to capture smoking status in risk factors if none is there (patients aged >15 years).
- Periodically run reports on patients due for screening or diabetic follow-ups.
- Enhance existing Diabetic Stamp to capture data in the EMR.
- Implement a diabetes prevention window showing summary of lab values.

#### Postengagement Evaluation

In this engagement, 2 baselines were collected—in March 2016, and again in June of that year after some initial data quality work. Owing to unforeseen factors (a new physician joined, entailing a data migration that interrupted the course of the engagement), the consultant was able to follow the practice for a longer period than is typical for the EPEP. Table 6 shows the improvement in data quality from the first baseline to the final assessment.

As this was one of the first EPEP engagements, this engagement provides the best picture of the sustainability of an EPEP-driven change. At the first assessment taken 6 months after the engagement began, we observed improvements in all areas; however, an even greater improvement in data quality is observed more than a year later, as the clinic had implemented recommendations and demonstrated their ability to sustain, and improve upon, the change.

Following the engagement, Dr. F reported increased efficiency in his clinic’s workflow and confidence in the quality of his EMR data. He believes he is seeing a benefit to his patients from these improvements. He encouraged other physicians in the group to engage with the EPEP, which they did.

**Table 6:** Postengagement data quality review—Dr. F.

Data quality indicator	Pre-engagement (January 2016)	First assessment (June 2016)	Postengagement (September 2017)
Breast cancer screening, %	66.00	67.00	76.00
Cervical cancer screening, %	59.30	62.00	69.00
Colorectal cancer screening, %	14.70	16.00	23.00
Smoking status captured, %	46.00	60.00	85.00
Patients with diabetes captured on the problem list^a^, n	201	226	231

^a^This indicator is reported differently in this engagement compared with the others; as one of the first Electronic Medical Record Practice Enhancement Program engagements, consultants were still refining the metrics they used for reporting and later revised the indicator to reflect those who were missing from the identified diabetes mellitus population, rather than those who were included.

## Discussion

### Principal Findings

As noted earlier in the paper, and born out in the literature, the EMR implementation alone does not guarantee proficiency— even with the passage of time [8,10]. Practices that engage in change management supports—including training, workflow analysis and corrections, resolution of data quality issues, and implementation of standards—are those that are most likely to realize a return on their digital health investment and improvement to patient outcomes [2,8,9,10,11]. The EPEP was designed to address these challenges, and consultants take a continuous quality improvement approach both in the context of engagement and in their practice and method.

In each case, we can see from pre- and postengagement DQR that improvements were achieved across most measures. These advances are further borne out by EPA assessments run at the baseline and postengagement for each practice. As noted in the Methods section, while the EPA is primarily a self-assessment tool, it is also used by EPEP consultants in the context of engagement—that is, EPAs are administered to determine a consultant/expert (rather than self) assessed maturity level. The resulting EPEP-assessed EPA dataset is separate from the larger self-assessed EPA cohort. At the time of writing, EPEP consultants had collected pre- and postengagement maturity assessments on over 200 completed engagements.

**Figure 3:**
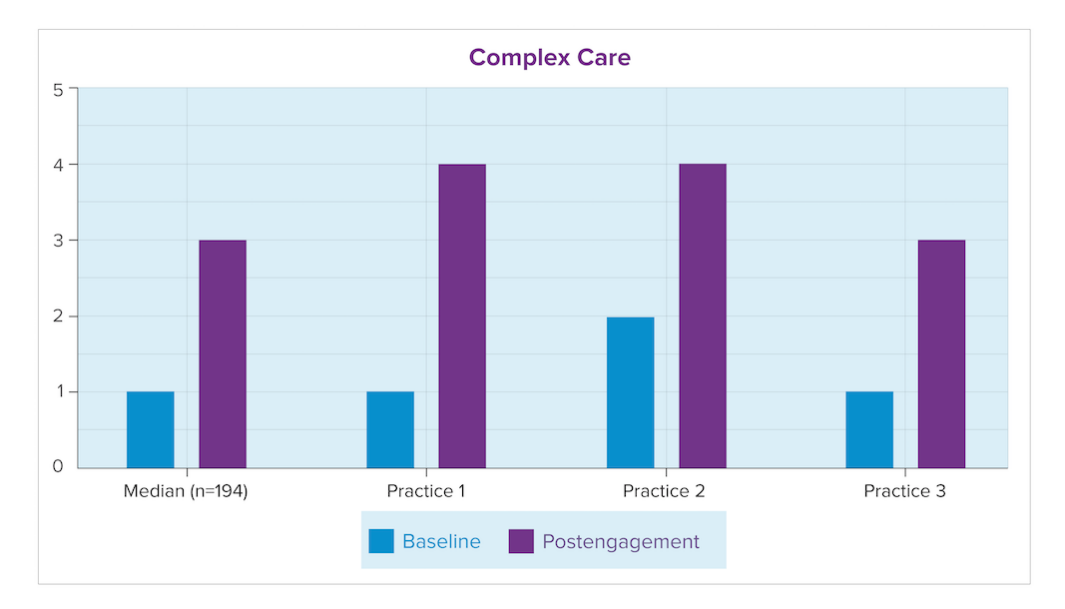
Maturity levels for Complex Care (includes chronic disease management).

**Figure 4:**
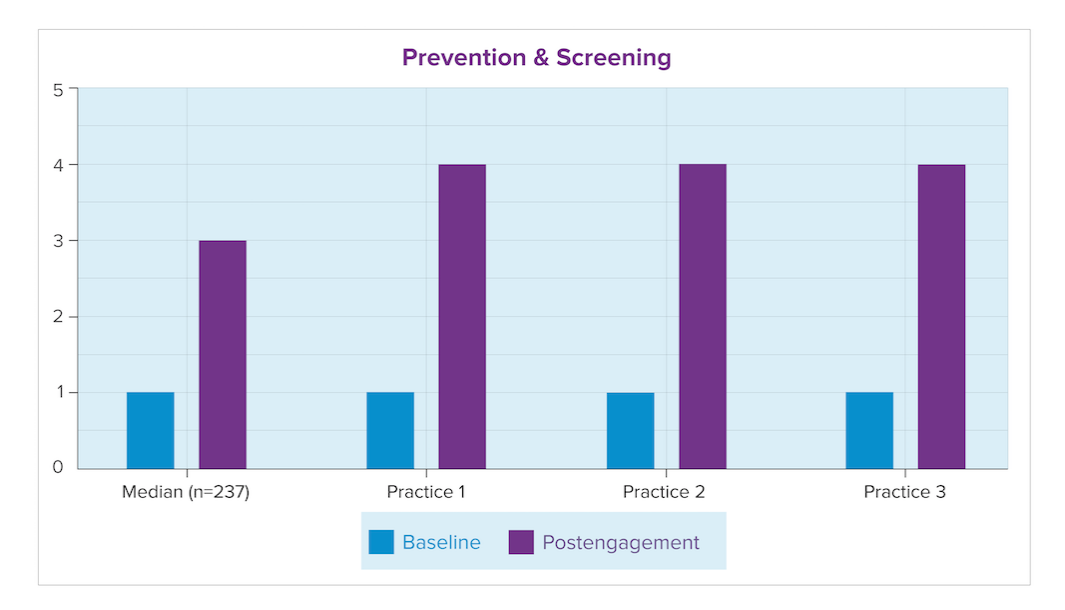
Maturity levels for Prevention and Screening.

In Figures 3 and 4, the baseline (current) maturity level for each practice here is shown to be similar to or higher than the median of the cohort of clinicians in this EPEP-assessed group (nb. the consultant-assessed *n* can differ between questions, as consultants use the EPA to assess only the areas a practice identifies as priorities). These figures show consultant-assessed maturity for each practice using the EPA, at baseline and postengagement, compared against the median of the entire cohort of consultant-assessed EPAs for the Complex Care key measure and Prevention and Screening key measure. At postengagement, 2 practices reported in this paper scored higher-than-median on their achieved proficiency for complex care; all 3 scored higher-than-median in prevention and screening.

### Limitations

The assessment approach used in this program, while mixed in its methods, relies heavily on human observation. It is thus vulnerable to subjectivity biases, but that potential limitation can be the price of applying expert interpretation to factors that influence the sustainability, or “stickiness,” of change.

With that qualification, several limitations could be expected to affect the reproducibility of results in applying an intervention of this nature. These include (1) variability in the nuances of executing the EPEP approach, across consultants; (2) variability of efficacy in implementing an action plan, across clinics (including supporting resources); and (3) variability in available functionality and supports, across EMRs.

An additional limitation concerns the EPA. As with the other assessment methods used in the EPEP, applying the EPA requires consultants to judge the level of proficiency they observe, which involves subjective as well as objective measurement. In the collective experience of EPEP consultants, movement up the maturity scale may be relatively straightforward with uncomplicated key measures like appointment scheduling. However, as for other key measures, like complex care and prevention and screening, gaining proficiency is more challenging, assessment of the maturity model may show very little movement from pre- to postengagement.

Interestingly, it is in these challenging-to-move key areas where, we believe, the value of the EPEP is most clearly demonstrated. Key measures where it is easier to advance—such as appointment scheduling—are more easily improved with use. In areas where improvement is more difficult to achieve, as noted at this paper’s outset, change management support, such as those delivered by the EPEP, can help a practice overcome these challenges, become more proficient, and sustain that level of proficiency.

### Implications for Future Analysis

Given these results, the next question might be—what factors lead to success in achieving EMR proficiency? Consultants routinely report that these successes are primarily because of the dedication of clinicians and staff at the practice, who embrace the process and understand that undertaking the recommended actions will result in tangible improvements in practice management and their capacity to provide quality care to their patients. Motivation to improve is a critical success factor. We note, for example, that at the baseline, Dr. A’s assessed maturity in complex care was a 1, but the desired level of maturity he reported for that measure was 5—considerably higher than the median. As has been found elsewhere [15], the knock-on effect of motivation to improve is the decision to seek supports—in this case, engagement with the EPEP—to achieve the desired changes.

Although the data presented here is limited to EPEP-assessed maturity, our collection of self-assessed EPA data (including measures matched across the EPA and its predecessor, the EMR Progress Report) now totals >1000 discrete respondents. From these data, we see a picture of steady progress in maturity across the province. As we continue to accrue macro-level data on the EMR maturity across Ontario, in combination with micro-level data from practice engagements, we will increasingly be able to characterize the factors that contribute to EMR proficiency and success at achieving quality improvement goals.

In the context of a large number of ongoing and future engagements for this relatively young program, these few examples cannot represent the program’s efficacy. Recognizing the importance of providing a clear account of the EPEP’s impact, consultants are routinely collecting pre- and postengagement data. As engagements accumulate, we will not only strengthen our ability to characterize the factors that contribute to EMR proficiency but also develop a better understanding of the EPEP’s impact, including the extent to which improved data quality and EMR proficiency postengagement correlates with better patient outcomes.

### Conclusions

The challenges described in these engagements are not unique to Ontario primary care practitioners. Technology adoption and implementation introduce disruption to clinical workflows, and the promise of a benefit may not be enough to embrace the change in a sustainable way fully.

The EPEP was established in recognition that to be sustainable, change requires support. Best practices in change management informed the EPEP method, and the program’s consultants operate as a team that regularly reflects on practice, shares new knowledge, and understands the value of consistency and rigor in their method and data collection.

With this customizable approach, EPEP consultants can virtually help any practice uncover gaps, achieve more efficient workflow, and improve data capture. Our previous analysis suggested that steps to improve EMR proficiency (maturity) can lead to improvements in care [16]. The examples described here add further layers to our understanding of EMR maturity—measurable improvements in data quality and ability to monitor patients can be achieved by individual practices as they work to improve EMR workflow and data quality. To be clear, while the achievement of objectives at the level of the practice is the goal, program consultants and clinicians involved in an engagement are serving system-level priorities as well. In Ontario, the Primary Care Performance Measurement Framework [17] identifies cancer screening, chronic disease (eg, diabetes) monitoring, and risk factor (eg, smoking) management as both system- and practice-level priorities for population health. As the EPEP program continues to spread and collect data from its engagements, we will be able to build a richer picture of the benefit of this change management approach for clinicians (practice-level) and the health system (system-level) alike.

The EMR use is a continuing journey of learning and improvement. Practitioners involved in our engagements have access to the supports necessary for sustainable change and continued progress. EMRs can improve population health management, enable public health interventions, and support evidence-based policy. Rather than focusing on the universal EMR adoption, resources should be aimed at moving the needle among existing EMR users to build capacity for better population health management. With appropriate help to improve EMR proficiency, practices can achieve their population health goals—to their patients’ benefit.

## Abbreviations

CPP: cumulative patient profile
DQR: data quality review
EMM: EMR Maturity Model
EMR: electronic medical record
EPA: EMR Progress Assessment
EPEP: EMR Practice Enhancement Program
HbA_1c_: glycated hemoglobin
MOHLTC: Ministry of Health and Long-term Care
